# Management of Congenital Methemoglobinemia in the Perioperative Setting: A Case Report and Review of Current Literature

**DOI:** 10.2147/JBM.S468072

**Published:** 2024-08-29

**Authors:** Moncef Ben Ghoulem Ben Saad, Arunabha Karmakar, Tayseer Salih Mohamed Salih, Wajeeha Arshad, Muhammad Jaffar Khan

**Affiliations:** 1 Department of Anesthesiology, Critical Care and Perioperative MedicineHamad Medical Corporation, Doha, Qatar

**Keywords:** methemoglobinemia, general anesthesia, perioperative management, cyanosis, methylene blue

## Abstract

**Background:**

Methemoglobin is an altered state of hemoglobin where iron in hemoglobin is oxidized and incapable of binding oxygen; leading to complications such as cyanosis, dyspnea, headache, and heart failure. Methemoglobinemia can be congenital or acquired. Congenital methemoglobinemia is a rare disease and its worldwide incidence is unclear. We recently encountered the first documented case of congenital methemoglobinemia at our institution, necessitating perioperative care.

**Case Presentation:**

In the present case, a 22-year-old man with congenital methemoglobinemia underwent general anesthesia for dental extraction. The surgeon was informed to avoid local anesthetics and oxygenation was performed with FiO_2_ of 1.0. Arterial blood gas analysis showed a PH of 7.337, PaO_2_ of 302 mm Hg, PaCO_2_ of 44 mm Hg, oxyhemoglobin level of 63.4%, and methemoglobin level of 37.8%. The patient had a stable course. No methylene blue therapy was required, although cyanosis was observed during surgery.

**Conclusion:**

In summary, though rare, congenital methemoglobinemia poses fatal risks during surgery. Its management involves preoperative recognition and optimization, oxygenation status, multidisciplinary care, avoiding precipitating or oxidizing agents, discussing treatment options, maintaining cardiopulmonary stability, and ensuring perioperative safety measures with the medical team.

## Introduction

Methemoglobin (MetHb) is an altered state of hemoglobin (Hb) containing iron in the Ferric (Fe^3+^) state rather than Ferrous (Fe^2+^). Ferric iron cannot bind and transport oxygen. Thus these patients can develop functional anemia and tissue hypoxia. MetHb is normally present in less than 1% of the blood concentration.^1^ With higher concentrations, symptoms develop.

Methemoglobinemia can be congenital and is usually rare. It can be attributed to either a cytochrome b5 reductase deficiency or the presence of hemoglobin M disease.^2^

Acquired methemoglobinemia is however more common and unfortunately more severe. This condition can arise from using different pharmacological agents including nitrates, and local anesthetics such as prilocaine, dapsone, and nitroglycerine (Box 1).^3^

We describe the perioperative management of a patient, diagnosed with congenital methemoglobinemia, who required general anesthesia for dental extraction.

**Box 1:** Common Agents Causing MetHb

**Chemicals**
Aniline dyes
Fava Beans
Fumes (Wood, Plastic, Automobile exhaust)
Ginkgo biloba
Herbicides
Mothballs
**Nitrates^a^**
Octane Boosters
Petrol
Well water
**Medications**
**Acetaminophen^a^**
Acetanilide
**Benzocaine^a^**
Bismuth subnitrate
Chloramine
Chloroquine
Copper sulfate
Dapsone
Flutamide
**Lidocaine^a^**
**Metoclopramide^a^**
**Nitric Oxide^a^**
Nitromethane
Nitrofurans
**Nitroglycerin^a^**
**Nitroprusside^a^**
Paraquat
Phenacetin
Phenazopyridine
**Phenytoin^a^**
**Prilocaine^a^**
Primaquine
Rasburicase
Silver nitrate
**Sodium valproate^a^**
Sulfasalazine
**Sulfonamides^a^**
Zopiclone

**Notes**: ^a^Common agent used in the operating room. Reprinted from Barash M, Reich KA, Rademaker D. Lidocaine-induced methemoglobinemia: A clinical reminder. *J Am Osteopath Assoc*. 2015;115(2):94–98. Creative Commons.^3^

## Case Report

### Background

We report a 22-year-old (98 kg bodyweight, 166 cm tall, BMI 35.6) Middle Eastern male, posted for elective extraction of carious wisdom teeth under general anesthesia. He was known to have congenital methemoglobinemia as well as some learning disability. Of note, two years prior, he underwent dental extraction in a dental clinic under local anesthesia, following which, he developed severe cyanosis later in his home, requiring hospital admission and intensive care unit management.

The patient also has had multiple previous visits to our hospital emergency department mainly with cyanosis and associated symptoms. Most of the ED visits had culminated in him receiving methylene blue and hydroxycobalamin before being discharged. A hematologist and cardiologist’s opinion had been documented in his health records. His co-existing learning disability suggested a diagnosis of Autosomal recessive congenital methemoglobinemia. Echocardiography had ruled out congenital cyanotic heart disease.

### Preoperative Assessment and Optimization

Armed with the available history, our focus was on the patient’s cardio-respiratory fitness. In the pre-anesthesia visit for his current surgery, our patient reassured us that he could comfortably engage in routine physical activities, including walking and climbing stairs, without experiencing headaches, palpitations, or dyspnea.

**Figure 1:**
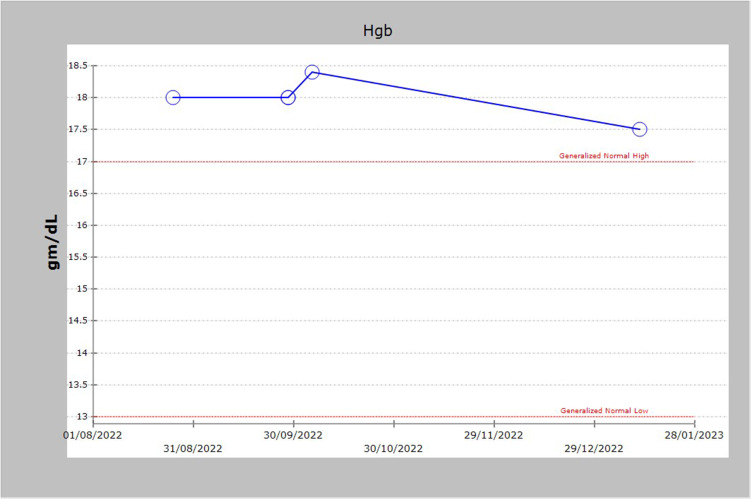
Patient had secondary polycythemia with Hb values ranging from 17.5 to 18.5 gm/dl.

**Figure 2:**
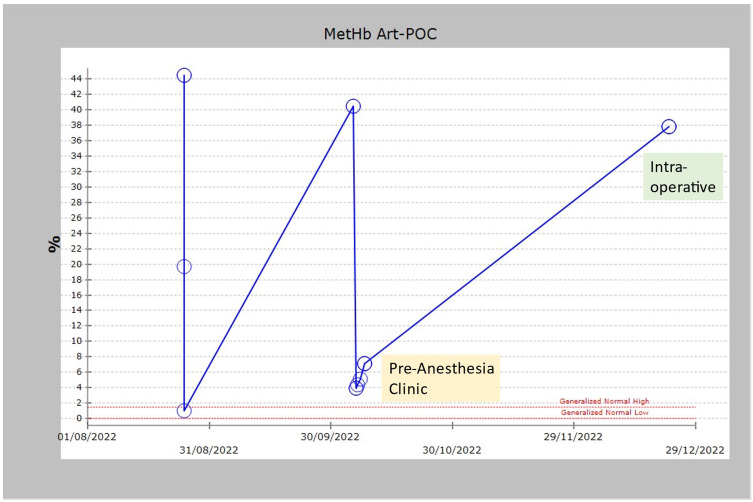
Patient had varying concentrations of MetHb with values from 1 to 44.5%.

On examination, he had visible bluish discoloration of his fingers and lips. The airway assessment revealed Mallampati grade II, adequate mouth opening, and normal neck movement. Oxygen saturation (SPO2) at the time was measured only 87% on room air. Blood investigations showed secondary polycythemia (Figure 1), Glucose-6-Phosphate Dehydrogenase (G6PD) within normal levels, and methemoglobin at around 3.9–7.1% (Figure 2).

A Transthoracic Echocardiography reported mild aortic regurgitation and trivial tricuspid regurgitation.

He was referred to a hematology and genetic clinic for whole exome sequencing.

The hematologist opined that the patient is fit for surgery and to avoid venesection for polycythemia as it will worsen his condition.

#### Intraoperative Management

On arrival in the operating room, baseline SPO2 was 84%. Following five minutes of preoxygenation with 100% oxygen, induction of general anesthesia was performed with calculated doses of fentanyl (2 mcg/kg), propofol (2mg/kg), and rocuronium (1mg/kg).

A 6.5 mm ID nasal endotracheal tube was inserted uneventfully and anesthesia was maintained with 100% oxygen, sevoflurane, and controlled ventilation (volume controlled, Tidal volume 450 mL, Respiratory rate 12/min, Peak airway pressure 23 mmHg, Positive end-expiratory pressure 5 mm Hg). SPO2 marginally improved to 87% on controlled ventilation and oxygenation.

An arterial line was inserted post-induction for frequent blood sampling for partial pressure of oxygen (PO2), patient acid-base balance, and methemoglobin level measurement. Invasive blood pressure monitoring was used to rapidly determine significant hemodynamic changes. The surgical team was requested to avoid lidocaine. Methylene blue was kept ready in case.

Methemoglobin level post induction was 37.8% and SPO2 was at 87% and considered inaccurate. The procedure was uneventful with no desaturation from baseline or acidosis in blood gases.

#### Postoperative Management

Emergence from general anesthesia and extubation was smooth. The patient was moved to the Post Anesthesia Care Unit (PACU) and admitted inpatient for continued observation.

## Discussion

Methemoglobin refers to a variant of hemoglobin that undergoes oxidation, leading to a transition in its heme iron configuration from the ferrous fe2+ to the ferric fe3+ state. This state of hemoglobin lacks oxygen-carrying capacity and in good health is normally present in less than 1% of the blood concentration (the normal range has been described as 1–3% in some sources).^1^

**Figure 3:**
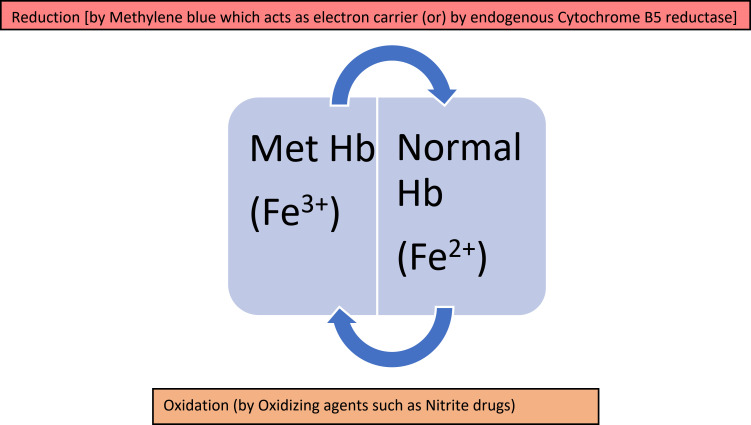
Equilibrium of Met Hb and Normal Hb in the body.

During red blood cell metabolism, methemoglobin is formed and converted back to its normal ferrous state at low levels. The process of methemoglobin production and reduction is typically balanced to uphold a steady-state level, which is around 1% of the total hemoglobin content (Figure 3).

Methemoglobinemia describes an imbalance in this equilibrium leading to Methemoglobin levels above the normal 1–3%. Hemoglobin deoxygenation, reactions with endogenous free radicals and exogenous chemicals can increase methemoglobin levels. Causes can be congenital or acquired (Table 1 and Box 1).^4^

**Table 1:** Causes of Congenital Methemoglobinemia

Autosomal Recessive	Autosomal Dominant
Cytochrome b5 reductase (CYB5R)deficiency (Congenital Methemoglobinemia)	Hemoglobin M disease
Subtypes: Congenital Methemoglobinemia Type I (enzyme defect in erythrocytes) Congenital Methemoglobinemia Type II (enzyme defect in all cells)	Subtypes: Boston Fort Ripley Hyde Park Iwate Kankakee Osaka Saskatoon

The failure of methemoglobin to effectively bind oxygen results in the inability to deliver oxygen to tissues. This condition presents a spectrum of symptoms and signs, ranging from simple cyanosis and the distinctive chocolate brown blood (10 to 20% MetHb concentration) to tachypnea, confusion, and syncope (MetHb concentration>30%).^4^ Central nervous system hypoxia with seizure and or coma, metabolic acidosis with dysrhythmia occurs for MetHb concentration more than 50% (Table 2).

**Table 2:** Symptoms and Signs in a Patient with Methemoglobinemia (Adopted from Ludlow et al).^2^

% MetHb	Symptoms
<15	Generally Asymptomatic
15–30	Cyanosis, anxiety, light-headedness, fatigue, headache
30–50	Tachypnea, confusion, syncope
50–70	Seizures, arrhythmias, metabolic acidosis, coma
>70	Death

Under normal physiological conditions, red blood cell enzyme cytochrome b5 reductase maintains low levels of methemoglobin. Hence, the primary cause of inherited methemoglobinemia is a congenital deficiency in cytochrome b5 reductase and is inherited in an autosomal recessive pattern.^2^ Hemoglobin M disease is the other form of congenital methemoglobin and is inherited as an autosomal dominant defect.^5^,^6^ In Hemoglobin M disease, a mutation in the gene coding for one of the globin chains results in a substitution of a tyrosine amino acid for either the proximal (F_8_) or the distal (E_7_) histidine amino acid in the α, β, or γ chains. This mutation stabilizes the iron in Fe^3+^ form. Most individuals with congenital methemoglobinemia show no symptoms apart from cyanosis.

An alternative pathway for methemoglobin reduction, which is not physiologically active, uses nicotinamide adenine dinucleotide phosphate NADPH methemoglobin reductase. NADPH is generated by glucose 6 phosphate dehydrogenase G6PD and this pathway is only activated by extrinsic acceptors such as methylene blue. This requirement of G6PD explains why Methylene Blue therapy is ineffective in individuals with G6PD deficiency.^7^

Congenital methemoglobinemia has three primary genetic causes. The most frequent cause is a deficiency in the CYB5R3 enzyme (autosomal recessive disorder). Following that, two other causes are hemoglobin M disease (autosomal dominant disorder) and cytochrome B5 deficiency. Although congenital methemoglobinemia due to cytochrome b5 reductase deficiency is exceptionally rare, but the actual incidence is unknown. Interestingly, it appears to occur more frequently among Siberian Yakuts, Athabaskans, Eskimos, and Navajo populations.^8^,^9^ These CYB5R3 deficiencies come in two types: type I, which affects only red blood cells, and type II, which occurs in all tissues. Patients with type I disease typically experience mild symptoms and have a normal lifespan. In contrast, those with type II disease exhibit cyanosis (bluish skin due to lack of oxygen), along with developmental delay, intellectual disability and other neurological manifestations, and have a significantly shorter lifespan.^1^,^10^

Acquired methemoglobinemia can range from severe to even potentially fatal depending on the plasma level. It can be a medical emergency and the diagnostic clues include cyanosis; respiratory or neurologic symptoms out of proportion to pulse oximetry; dark red brownish to blue blood that does not turn red with oxygenation; and low pulse oximetry that does not improve with oxygen. These symptoms generally occur with a methemoglobin level of 10% and a level of more than 30% can be life-threatening. Acquired methemoglobinemia may be triggered by various oxidizing agents such as dapsone, chloroquine, metoclopramide, benzocaine, lidocaine, prilocaine, nitric oxide, nitroglycerin, and others (Box 1).^3^,^11–13^

Methylene blue is often administered (1–2mg/kg IV) to treat patients with methemoglobinemia however it should be avoided in patients with G6PD deficiency due to the risk of hemolysis. A previous study found that methylene blue did not improve methemoglobinemia in patients with Hb M.^14^ Therapy with vitamin C can be considered when methylene blue is not indicated. Prophylactic preoperative methylene blue administration in a patient with congenital MetHb lowered the methemoglobin level significantly. This led to a notable rise in oxygen saturation, providing a greater safety margin against hypoxemia during the perioperative period. Alternatively, Hyperbaric oxygen (HBO) therapy was found an effective treatment in MetHb. HBO therapy inhibits the oxidation of hemoglobin by nitrite and reduces MetHb levels by approximately 8% per hour.^1^,^15^

**Figure 4:**
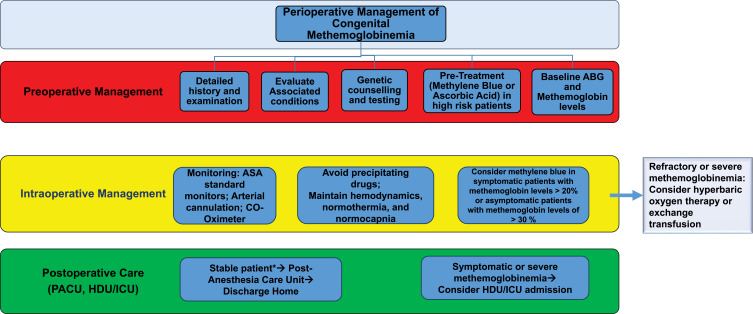
Recommendations for Perioperative Management of Congenital Methemoglobinemia.

**Notes**: *Stable patient refers to an asymptomatic, hemodynamically stable patient. Such patients can be monitored in the PACU with frequent measurement of methemoglobin levels and standard care.

Inhalation of high concentrations of oxygen can be also used to treat methemoglobinemia and high arterial oxygen pressure should be maintained to minimize tissue hypoxia during induction of general anesthesia. Additionally, blood transfusion or exchange transfusion should be considered in patients with severe MetHb exceeding 70%.^6^,^16^,^17^ A Summary of recommendations for perioperative management of congenital methemoglobinemia is given in Figure 4.^18^

There have been few reported cases of perioperative management of congenital methemoglobinemia in the literature. In this report, we have also summarized all the reported cases of congenital methemoglobinemia and anesthesia management (Table 3).

**Table 3:** Summary of Reported Cases of Perioperative Management of Congenital Methemoglobinemia

Author	Age/Sex	Associated Conditions	Surgery	Anesthetic Management	Postoperative course	Outcome
Chisholm et al^19^	24 years old/ Female	Nil	Evacuation of retained product of conception	Anesthesia was induced with intravenous propofol and fentanyl and maintained with 50% nitrous oxide in oxygen with incremental propofol boluses.	Uneventful	Discharged home the next day
Baraka et al^20^	22 years old/ Male		Turbinectomy	Arterial cannula inserted, then lidocaine (1mg/kg) was administered intravenously. The patient developed sudden unconsciousness, and apnea, with severe cyanosis and desaturation (79%). 100% oxygen and methylene blue (1mg/kg) were administered. The patient regained consciousness but the surgery was cancelled.	Stable course	Not reported
Maurtua et al^21^	33 years old/ Female	Nil	Laparoscopic excision of a right rudimentary fallopian tube and hysteroscopy	Arterial line was inserted, followed by intravenous induction with fentanyl, lidocaine, propofol and atracurium	Stable perioperative course, Arterial blood gas (ABG) taken in the recovery room showed no change in MetHb in levels from the baseline.	Not reported
Baraka et al^22^	26 years old/ Male	Nil	Turbinectomy	Arterial line with baseline blood gas sampling, prophylactic methylene blue 1% administered at a dose of 1mg/kg intravenously, followed by IV induction with lidocaine (1mg/kg), propofol (2mg/kg) and rocuronium (0.6mg/kg). Anesthesia was maintained with isoflurane 1 to 2% in 50% of oxygen.	Uneventful course. MetHb fraction was 0.01 postoperatively and increased to 0.026 on the second day to reach 0.094 on the fifth day.	Not reported
Sharma et al^23^	9 years old/ Male	Osler-Weber-Rendu syndrome and bilateral pulmonary arteriovenous malformation	Cerebral angiography	Radial artery cannulated, followed by intravenous (IV) induction with thiopentone sodium (150 mg), and rocuronium (25 mg). Inhalational anesthesia was maintained with 0.8–1% isoflurane. Reversed with IV neostigmine 1.5 mg and atropine 0.6 mg before extubation.	Stable postoperative period.	Not reported
Melarkode et al^14^	60 years old/ Female	Nil	Mastectomy	Arterial line was inserted before anesthesia, induction with IV fentanyl and propofol. Inhalational anesthesia was maintained with Isoflurane.	Uneventful perioperative course	Discharged home two days after the surgery.
Lin et al^6^	35 years old/ Female	Nil	Uterine myomectomy	Following preoxygenation with 100% oxygen, anesthesia was induced with IV midazolam (2.5 mg), fentanyl (75 mcg), lidocaine (50 mg), propofol (2mg/kg) and rocuronium (50 mg). Extubated at the end of the surgery	Smooth recovery.	Discharged home two days later.
Champigneulle^24^	78 years old/ Male	Nil	Aortic valve replacement	General anesthesia was induced with the target-controlled infusion of propofol and remifentanil and a bolus of atracurium.	Extubated a few hours after the surgery.	Discharged from the ICU on day 2 and from the hospital on day 13 without complications.
Gupta et al^25^	26 years old/ Female	Nil	Emergency cesarean section	Spinal anesthesia with 0.5% bupivacaine (2mL).	Stable postoperative course.	Not reported
Yin et al^26^	22 years old/ Female	Nil	Elective induction of Labor	Remifentanil as intravenous labor analgesia, at an infusion of 0.025 mcg/kg/min with a 25 mcg bolus and continuous methemoglobin saturation (CO-Oximeter) monitoring.	Smooth recovery	Discharged home on postpartum day 2.
Ri et al^4^	32 years old/ Male	Hb M disease	Thyroidectomy	Arterial line was inserted followed by IV induction with propofol (200 mg) and rocuronium (90 mg). Anesthesia was maintained with 1.5–2% sevoflurane with FiO2 at 0.6. Target remifentanil set at 2–3 ng/mL IV. Frequent ABG samples taken intraoperatively. Twenty minutes post-induction, SpO2 was 65–75% and cyanosis was observed. FiO2 increased to 1.0. Extubated after two hours of surgery.	Received oxygen at 6 L/min via face mask in the recovery and weaned to room air later.	Uneventful postoperative course and discharged home on day 5 after surgery.
Choi et al^27^	15 years old/ female	Nil	Dental extraction	Remifentanil and propofol infusion started at 0.5 mcg/kg/hr and 100 mcg/kg/min respectively to facilitate arterial cannulation, followed by an induction dose of propofol (150 mg) without neuromuscular blockade. Anesthesia was maintained with isoflurane inhalation and IV remifentanil. CO-Oximeter used intraoperatively for continuous measurement of MethHgb along with cerebral oximetry. Received 4 mg each of dexamethasone and ondansetron as well as 50 mcg of fentanyl intraoperatively. Extubated uneventfully at the end of the surgery	Placed on 6 L/min of oxygen by the face mask and monitored for four hours postoperatively.	Discharged home on the same day of surgery
Karimbanakkal et al^28^	Middle-aged, Male	Diabetes, hypertension, coronary artery disease, post-renal transplant	Parathyroidectomy	After preoxygenation with 100% oxygen, induced with IV etomidate (16 mg), fentanyl (100 mcg), and atracurium (40 mg). Anesthesia was maintained with sevoflurane and IV fentanyl 50 mcg/hr. Arterial line inserted post-induction. Extubated after given reversal. Maintained stable hemodynamic throughout the surgery	Shifted to intensive care unit post-operatively and placed on 4–6 L/min oxygen via Hudson mask.	Not reported

In our patient MetHb concentration was 37.8% and pH 7.337, PaO2 302 mm Hg, PaCO2 44 mm Hg, and oxyhemoglobin level of 63.4%. Oxygenation and ventilation with FiO2 at 100% were maintained and the intraoperative vital signs such as blood pressure and heart rate remained stable. Repeated blood gases were taken during the procedure to follow MetHb level and acid-base balance to decide methylene blue utilization (first line).

The routine use of local anesthetic administration was prohibited and no Methylene Blue therapy was needed. Postoperatively, the MetHb level was still 37.8%. We admitted the patient for 24 hours for post-operative observation. He remained stable and was discharged home a day after the procedure. Genetic testing later revealed homozygosity for the cytochrome b5 reductase-3 enzyme leading to cytochrome b5 reductase deficiency.

## Conclusion

In conclusion, although congenital methemoglobinemia is rare, it is still life-threatening and therefore warrants strong perioperative consideration. Patients with congenital or inherited methemoglobinemia pose a challenge with respect to perioperative management of oxygenation and global tissue perfusion.

The avoidance of potential oxidizing agents as well as the availability of emergency treatment modalities such as methylene blue or exchange transfusion and hyperbaric oxygen therapy should be discussed. A card with a list of contraindicated medications could be given to the patient to prevent the patient’s future exposure and disease exacerbation.

Intraoperatively the anesthesiologist should discuss the safety precautions with the nursing and surgical team. The use of oxidizing agents must be avoided and oxygen carrying capacity must be maintained by ensuring high oxygen concentration in the inhaled gases. Methylene blue can be given prophylactically or as therapy, if the patient has a normal G6PD level. The decision to treat or not is ideally guided by clinical judgment and serial blood gases to check MetHb level, oxyhemoglobin, and acid-base balance can be used to support clinical findings. Blood transfusion or exchange transfusion can also be considered. A hyperbaric oxygen therapy chamber within the hospital facility might also be a great advantage.
